# Quantum Mechanical Predictions of the Antioxidant Capability of Moracin C Isomers

**DOI:** 10.3389/fchem.2021.666647

**Published:** 2021-04-21

**Authors:** Angela Parise, Bruna Clara De Simone, Tiziana Marino, Marirosa Toscano, Nino Russo

**Affiliations:** ^1^Dipartimento di Chimica e Tecnologie Chimiche, Università della Calabria, Rende, Italy; ^2^Université Paris-Saclay, CNRS, Institut de Chimie Physique UMR8000, Orsay, France

**Keywords:** moracin, antioxidants, DFT, kinetic constants, reaction mechanisms

## Abstract

The antioxidant capability of moracin C and *iso*-moracin C isomers against the OOH free radical was studied by applying density functional theory (DFT) and choosing the M05-2X exchange-correlation functional coupled with the all electron basis set, 6-311++G(d,p), for computations. Different reaction mechanisms [hydrogen atom transfer (HAT), single electron transfer (SET), and radical adduct formation (RAF)] were taken into account when considering water- and lipid-like environments. Rate constants were obtained by applying the conventional transition state theory (TST). The results show that, in water, scavenging activity mainly occurs through a radical addition mechanism for both isomers, while, in the lipid-like environment, the radical addition process is favored for *iso*-moracin C, while, redox- and non-redox-type reactions can equally occur for moracin C. The values of pKa relative to the deprotonation paths at physiological pH were predicted in aqueous solution.

## Introduction

In the last decades, 2–phenyl–benzofuran-containing molecules, found in a variety of plants (*Morus alba, Artocarpus champeden, Erythrina addisoniae, and Calpocalyx dinklagei*) (Hakim et al., 1999; Na et al., 2007; Naik et al., 2015; Kapche et al., 2017; Pel et al., 2017), have attracted considerable interest both for their massive use in pharmacology and for their ancient use in traditional medicine in Asia, Africa, and America (Fashing, 2001; Venkatesh and Seema, 2008; Kapche et al., 2009; Kuete et al., 2009). A rich source of natural products with a 2–phenyl–benzofuran basic scaffold is the *Moraceae* family (e.g., *M. alba, Morus mesozygia, Morus lhou*, and *Morus macroura*) (Sang-Hee et al., 2002), from which more than 24 molecules (moracin A–Z) have already been isolated and characterized (Nguyen et al., 2009). Many of them showed a variety of biological and pharmacological activities and were tested as potent antioxidants (Kapche et al., 2009; Seong et al., 2018), anti-cancer agents (Nguyen et al., 2009), anti-inflammatories, and anti-microbial agents (Kuete et al., 2009; Zelová et al., 2014; Lee et al., 2016). Furthermore, they were proven to act as cholinesterase (Delogu et al., 2016; Seong et al., 2018) and β-site amyloid precursor protein cleaving enzyme 1 (BACE1) (Jeon et al., 2007; Seong et al., 2018) inhibitors *in vitro*.

**Figure 1:**
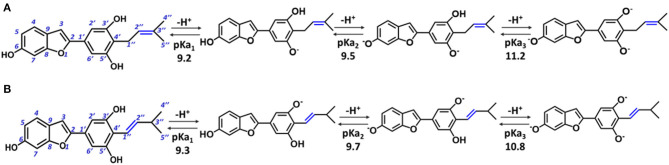
Structure of moracin C and *iso*-moracin C, and pKa values of the relative deprotonation paths at physiological pH. **(A)** Moracin C. **(B)** Iso-moracin C.

In particular, moracin C {2–[3′,5′-dihydroxy−4′-(3–methylbut−2–enyl)phenyl]−6–hydroxybenzofuran} and its *iso*–moracin C isomer {2–[3′,5′-dihydroxy−4′-(3–methylbut−1–enyl)phenyl]−6–hydroxybenzofuran} (see Figure 1), extracted from *M. alba* and *Artocarpus heterophyllus*, exhibit antioxidant capabilities (Li et al., 2018; Seong et al., 2018) and other biological functions correlated with oxidative stress (Zelová et al., 2014; Naik et al., 2015; Li et al., 2018; Seong et al., 2018). The only structural difference between the two isomers is the position of the C=C double bond in the methylbut–enyl moiety (see Figure 1). This apparent small structural difference may have significant consequences on the electronic and reactivity properties of the two isomers. In fact, when the C=C bond is close to the phenyl ring (as occurs in *iso* moracin), the electronic delocalization between the two groups increases, stabilizing accordingly the radical that is formed as a result of O–H abstraction reaction. On the contrary, the localization of the double bond in position 2″ prevents conjugation with the phenolic ring and, in principle, would favor radical attack reactions.

Very recently, in an accurate experimental study (Li et al., 2018), the authors attempted to correlate the estimated antioxidant properties with the position of the C=C bond in the two isomers, concluding that “[B]oth moracin C and *iso*–moracin C can inhibit ROS, likely through redox-related pathways (especially ET and H^+^-transfer) and a non-redox-related RAF pathway. In the redox-related pathways, a double bond at the conjugation position can enhance the ET and H^+^-transfer potential. However, in the non-redox-related pathway, the double bond position hardly affected the RAF potential.”

We have conducted an accurate theoretical study on the thermodynamic and kinetic properties of moracin C and *iso*-moracin C when reacting with the OOH free radical by considering the following most common antioxidant scavenging reaction mechanisms (Leopoldini et al., 2011; Alberto et al., 2013; Mazzone et al., 2015; Galano et al., 2016; Markovic et al., 2016; Ahmadi et al., 2018; Castaneda-Arriaga et al., 2020; Romeo et al., 2020):

HAT: hydrogen atom transfer

➢ H_3_X + R^·^ → H_2_X^·^ + RH
➢ H_2_X^−^ + R^·^ → HX^−·^ + RH

RAF: radical adduct formation

➢ H_3_X + R^·^ → [H_2_X ^__^RH]^·^
➢ H_2_X^−^ + R^·^ → [HX^__^ RH]^−·^

SET: single electron transfer

➢ H_3_X + R^·^ → H_3_X^+·^ + R^−^
➢ H_2_X^−^ + R^·^ → H_2_X^·^ + R^−^

## Computational Details

All calculations were performed with the Gaussian 09 code (Frisch et al., 2009) by applying the density functional theory. Following a well-consolidated protocol that was proven to be reliable in a large amount of antioxidant systems (Galano et al., 2016; Pérez-González et al., 2020), the M05-2X functional (Zhao et al., 2006) and the all electron basis set, 6-311++G(d,p) were chosen for all computations. Geometry optimization without any constraint was followed by frequency calculations to verify if the obtained structures were local minima (0 imaginary frequency) and transition states (TSs) (1 imaginary frequency) and to obtain zero-point energy corrections. Furthermore, for the TSs, it was verified that the imaginary frequency matched with the expected motion along the reaction coordinate. The solvation model based on density (SMD) (Marenich et al., 2009) was used to mimic the aqueous and lipid-like environments (water and pentyl ethanoate, respectively). Intrinsic reaction coordinate computations were performed to verify if the intercepted TSs properly connected to the relative minima in a given path.

Relative energies were computed with respect to the sum of separate reactants, and the thermodynamics corrections at 298.15 K were taken into account following the quantum mechanics-based test for the overall free radical scavenging activity (QM-ORSA) procedure (Galano and Alvarez-Idaboy, 2013, 2019). Rate constants, *k*, were determined by applying the conventional transition state theory (TST) at the 1M standard state (Truhlar et al., 1996). For the mechanism involving SETs, the barriers of reaction were computed using the Marcus theory (Marcus, 1957). For rate constants, close to the diffusion limit, the Collins–Kimball theory (Collins and Kimball, 1949) was applied.

## Result and Discussion

For the study in water environment, knowledge of the acid-base equilibria under physiological conditions (pH = 7.4) is very important. Because of lack of experimental information on both studied isomers, the relative pKa values were obtained (Table 1) using the parameters fitting method, which was previously proven to give results that are in good agreement with experimental data (Pérez-González et al., 2018). The deprotonation path of the two study systems is shown in Figure 1. The preferred deprotonation site in moracin C is the OH in the C5′ position, followed by those in C6 and C3′. On the contrary, in *iso*-moracin C, the preferred deprotonation site is the OH in the C3′ position, while the second and the third ones involve sites C6 and C5′, respectively. In both conformers, all deprotonation sites are found in the benzene ring. A look at the molecular electrostatic potential, whose maps are reported in Supplementary Figure 1, shows that, in the case of *iso*-moracin C, the presence of the double bond in position C1′-C2′ increases the π delocalization as proven by a great negative charge on the oxygen of the hydroxyl group on C5′ position. The charge distribution reported in Supplementary Table 1 further underlines that the localization of the double bond of methylbut–enyl moiety can influence the acid–base equilibrium of the two isomers. The calculated pKa values, at pH = 7.4 (see Table 1), indicate that, for both isomers, the neutral species are prevalent (molar fractions are 0.98 and 0.99 for moracin and *iso*-moracin, respectively). The monoanionic forms were not negligible in both isomers (see Supplementary Figure 2), so the H_3_X and H_2_X^−^ species were considered in the water environment study.

**Table 1:** pKa value and molar fractions (Mf) of the different acid–base species of moracin C and *iso*-moracin C, at physiological pH.

**Molecule**	**pKa_**1**_**	**pKa_**2**_**	**pKa_**3**_**	**Mf (H_**3**_X)**	**Mf (H_**2**_X)^**−**^**	**Mf (HX)^**2−**^**	**Mf (HX)^**3−**^**
Moracin *C*	9.2	9.5	11.2	9.8 × 10^−1^	1.6 × 10^−2^	1.2 × 10^−4^	2.0 × 10^−8^
Iso-moracin *C*	9.3	9.7	10.8	9.9 × 10^−1^	1.2 × 10^−2^	6.2 × 10^−5^	2.5 × 10^−8^

**Figure 2:**
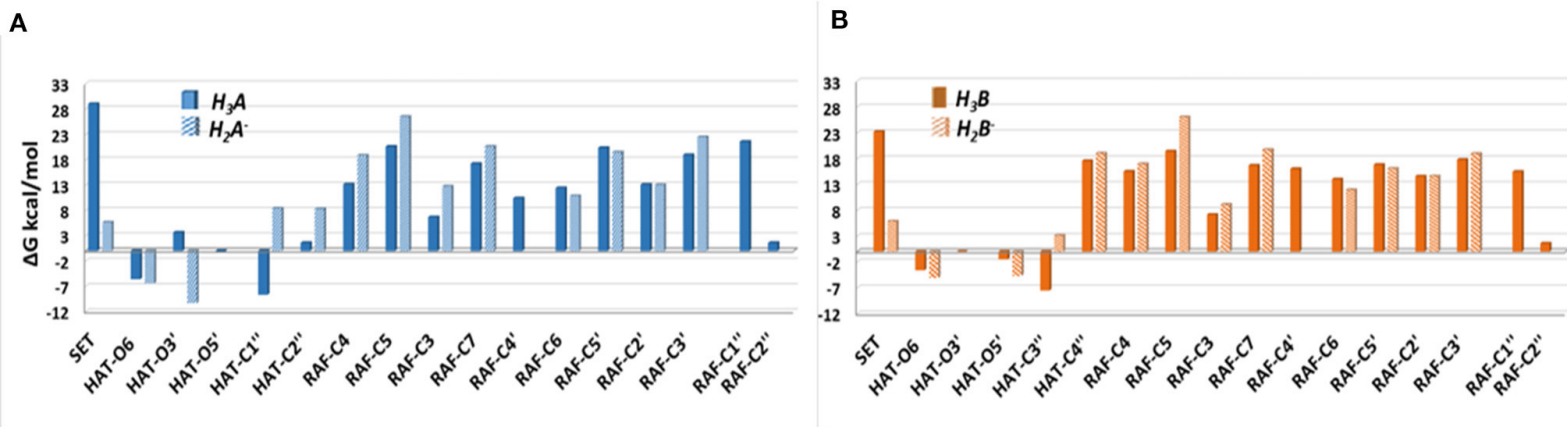
Relative Gibbs free energies (ΔG kcal/mol) values at 298.15 K for neutral moracin C (H_3_A), monoanion (H_2_A^−^), neutral *iso*-moracin C (H_3_B), and monoanionic (H_2_B^−^) species in aqueous solution. **(A)** Moracin C. **(B)** Iso-moracin C.

**Figure 3:**
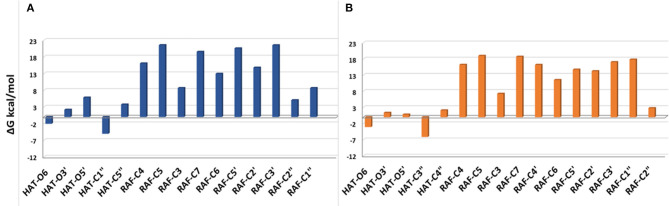
Gibbs free energies of reaction (ΔG kcal/mol) at 298.15 K for neutral moracin C (H_3_A) and *iso*-moracin C (H_3_B) in pentyl ethanoate solvent. **(A)** Moracin C. **(B)** Iso-moracin C.

The Gibbs free energies of reaction (ΔG), computed for the two investigated mechanisms in water and lipid-like environments, are reported in Figures 2, 3. As can be seen, for both molecules and environments, ΔG values for the RAF mechanism are all positive. However, since a recent experimental study (Li et al., 2018) suggested that this kind of mechanism might happen instead, we have also considered the addition of the OOH free radical to the C2″ sites, in which the obtained Gibbs reaction energies assume the less positive values.

Although the ΔG values obtained for SET are always positive, we have also considered this mechanism that was found active in several systems that had been previously studied (Galano et al., 2016; Castaneda-Arriaga et al., 2020; Romeo et al., 2020). From Figure 2, it is clear that HAT in the aqueous solution occurs preferentially at C1″, O6, and O5′ sites of the moracin C neutral form and O6 and O3′ sites of the corresponding monoanion. For *iso*-moracin, HAT is favored at C3″, O6, O5′, and O3′ sites of the neutral form and at O6 and O5′ sites of the monoanion one.

In the pentyl ethanoate solvent, where only the neutral species are present, the HAT process is favored, and the lowest ΔG values are obtained for the OOH attack at the C1″ site followed by O6 for moracin C and at C3″ and O6 for *iso*-moracin C.

**Figure 4:**
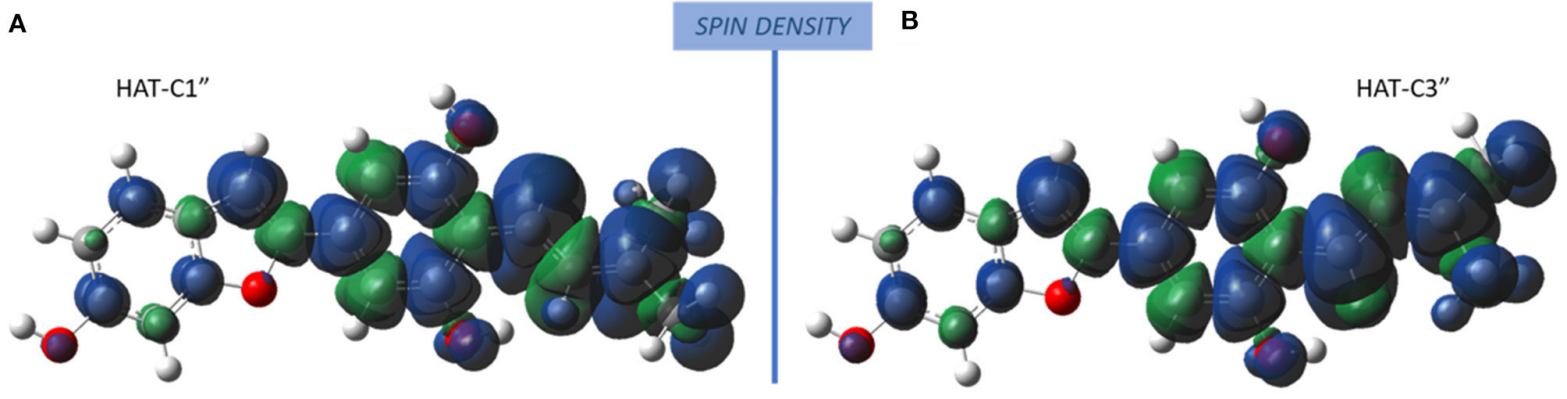
Spin density distribution in the radical obtained after the HAT process at C1″ (moracin) and C3″ (*iso*-moracin) sites. **(A)** Moracin C. **(B)** Iso-moracin C.

The radicals obtained following the abstraction of the proton by OOH free radical have a spin density that is distributed over almost the entire molecular structure, as it is reported in Figure 4 that the spin density plots of moracin C deprotonated on C1″ and *iso*-moracin C deprotonated on C3.″ In particular, due to the C=C double bond proximity to the phenyl ring, electron delocalization appears to be slightly more extended in *iso*-moracin C. In any case, this trend suggests good stability of the formed radical species for both molecules.

**Figure 5:**
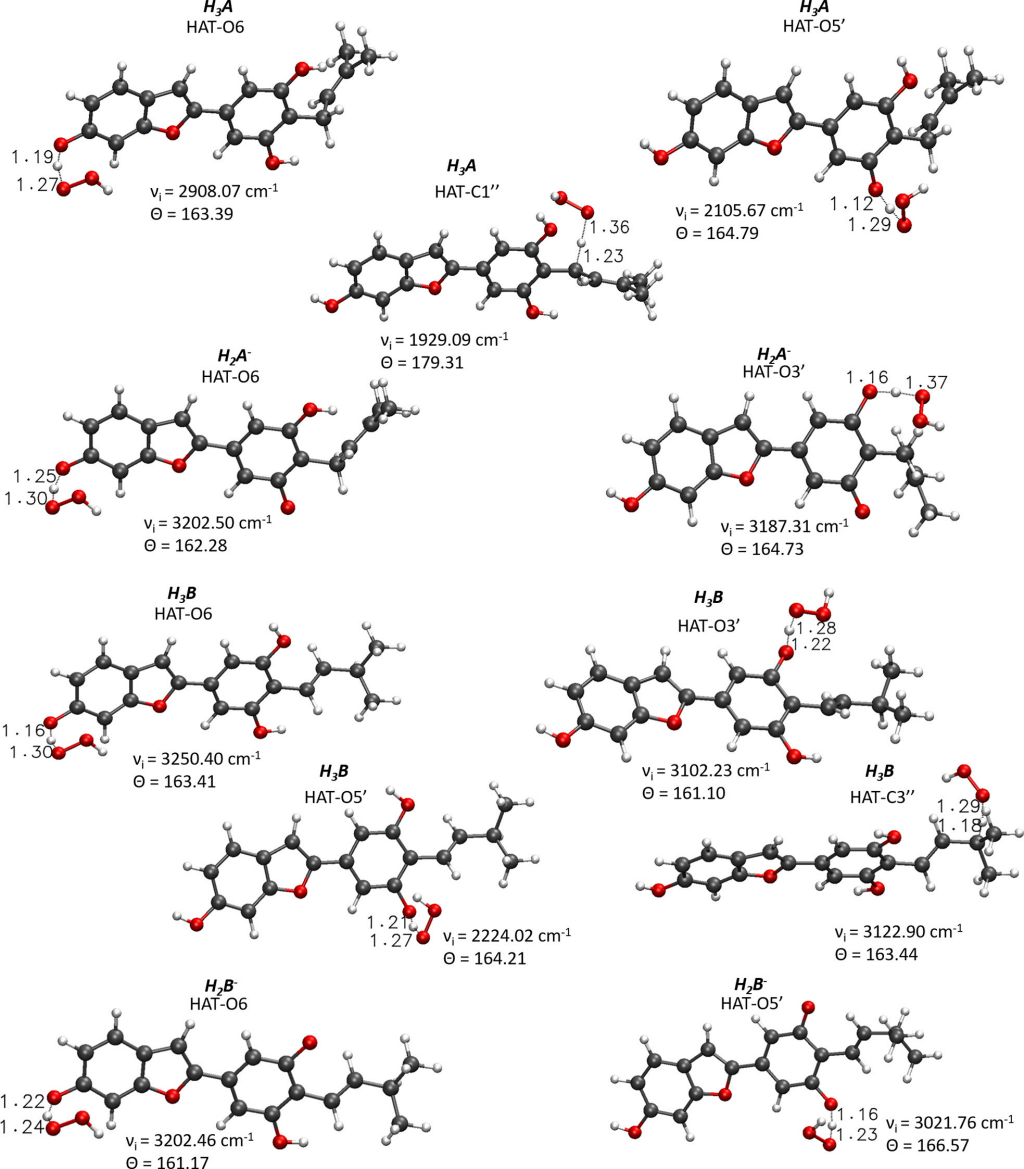
Main geometrical parameters for the optimized TSs structures for the neutral and monoanionic species of moracin C and *iso*-moracin C involved in the HAT mechanism. Bond lengths are in Å, angles in degrees, and imaginary frequencies in cm^−1^.

For the processes that show exergonic, almost isergonic, and moderate endergonic behaviors, we have computed the kinetic constants. To do this, it was necessary to locate the TSs to obtain the activation energies for the given reaction mechanism. The structures of all TSs obtained for the HAT process and their relative imaginary frequencies are shown in Figure 5 for neutral and monoanionic species in the aqueous environment, while the structures of TSs of the neutral systems in the lipid-like environment are shown in Supplementary Figure 3. The obtained energy barriers (ΔG^#^) are reported in Table 2 together with the Gibbs free energies of reaction.

**Table 2:** Gibbs free energies of reaction (ΔG) and activation (ΔG^‡^ kcal/mol) at 298.15 K in aqueous solution for neutral and monoanion moracin C and *iso*-moracin C species for the considered mechanisms.

**Mechanism**	**H_**3**_A**		**H_**2**_A^**−**^**		**H_**3**_B**		**H_**2**_B^**−**^**		**H_**3**_A^**PE**^**		**H_**3**_B^**PE**^**	
	**ΔG**	**ΔG^**‡**^**	**ΔG**	**ΔG^**‡**^**	**ΔG**	**ΔG^**‡**^**	**ΔG**	**ΔG^**‡**^**	**ΔG**	**ΔG^**‡**^**	**ΔG**	**ΔG^**‡**^**
SET	29.03		5.65		23.21		5.89					
HAT-O6	−5.62	19.78	−6.43	19.81	−3.62	21.04	−5.12	19.52	−0.02	17.23	−3.05	15.39
HAT-O3′	3.63		−10.32	17.04	0.01	19.86					0.84	17.23
HAT-O5′	−0.14	20.94			−1.81	26.27	−4.71	18.15				
HAT-C1″	−8.66	19.06							−5.02	6.08		
HAT-C3″					−7.52	17.85					−6.12	11.95

*Apex PE refers to the neutral moracin C and iso-moracin C in pentyl ethanoate solvent*.

Inspection of the last Table reveals that, in aqueous solution, the ΔG^#^ values of moracin C fall in the range of 19–21 kcal/mol for the neutral forms and become slightly lower for the charged ones. A similar behavior can be noted for *iso*-moracin C species. In the pentyl ethanoate solvent, the result is different, and for some sites, the barriers are sensibly smaller (e.g., 6.08 kcal/mol for the C1″ site). We would like to underline that the C–H bonds of the 3–methlbut−2-enyl in moracin C and in *iso*-moracin C are involved in the HAT process, making these two natural molecules interesting antioxidant agents.

**Figure 6:**
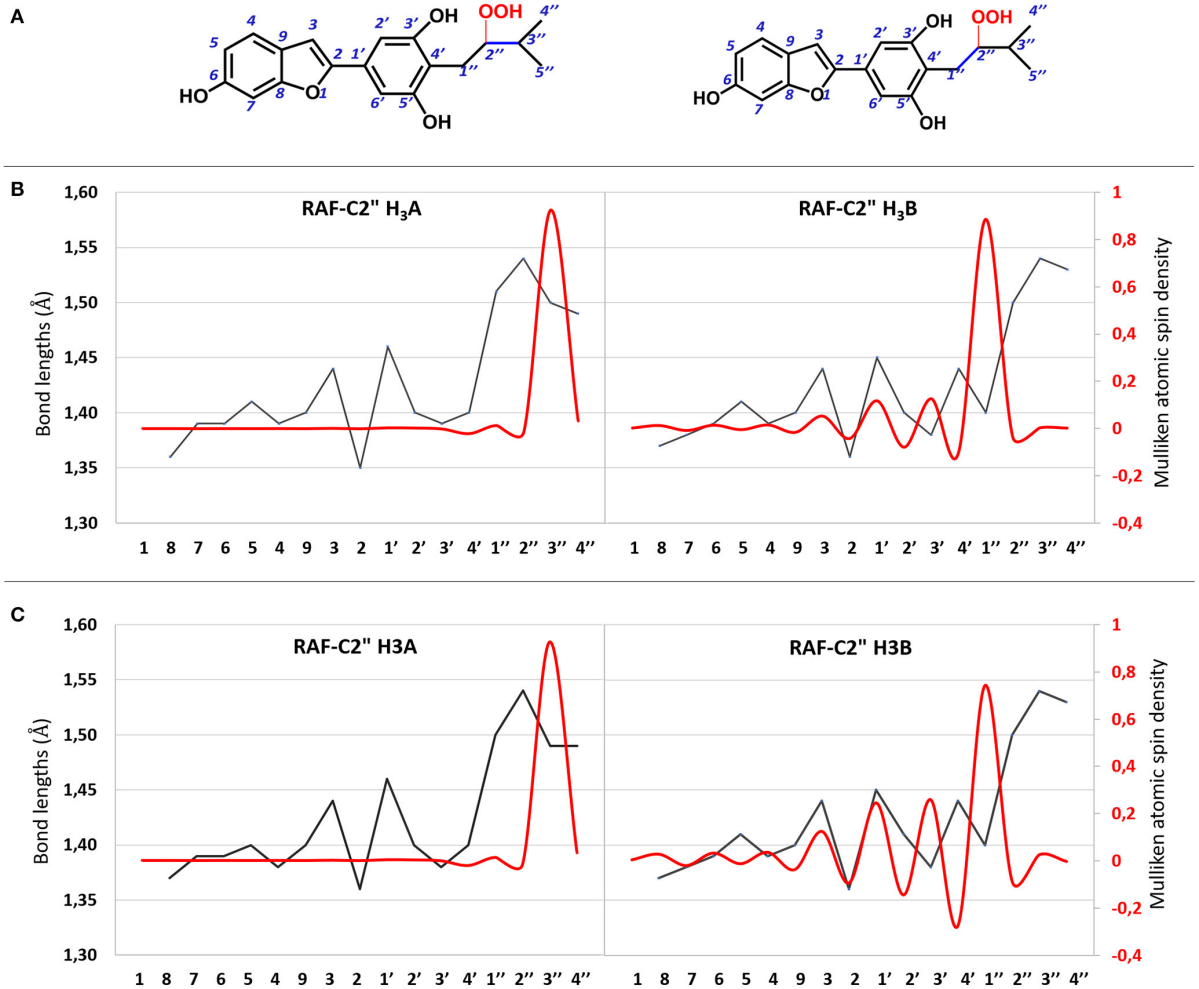
**(A)** 2D representation of OOH addition to the C2″ atom of moracin C and iso-moracin C. **(B)** CC bond lengths (black line) and atomic spin density (red line) for the OOH addition to the C2″ atom in water enviroment. **(C)** CC bond lengths (black line) and atomic spin density (red line) for the OOH addition in PE environment.

All attempts to locate the relevant TS for the radical attack to the C2″ site for both molecules failed. However, this is not unusual since this type of radical attack often occurs without energy barriers. The structures derived from the OOH radical attack on the C2″ atom for both molecules are reported in Supplementary Figure 4. The C=C bond variation and atomic spin density for the moracin C–OOH and *iso*-moracin C–OOH radical adduct in both considered environments are shown in Figure 6, and the corresponding values are reported in Supplementary Table 2. An inspection of Figure 6 shows that the addition of the OOH radical on the C2″ atom induces different effects in the two tautomers. In fact, in moracin C, in both the considered solvents, the spin density is essentially located at C3″ and the bond length results of C2″-C3″ needs to be elongated by assuming values close to those of a single bond (1.513 and 1.491Å in water and PE, respectively). In *iso*-moracin C, in the water environment, the addition of the radical in the same position induces a large spin density in the C1″ atom, a smaller but significant one in C3′ and C1′ atoms, and a very small density in C3 and C4 atoms. The C2″-C1″ distance is now 1.504 Å. This means that this radical, with a more extended spin density distribution result, would be more stable than the corresponding in moracin C. Similar relationships have previously been observed in other theoretical investigations on the antioxidant power of carotenoid derivatives (Ceron-Carrasco et al., 2010).

The computation of the electrodonating (ω^−^) and electroaccepting (ω^+^) values, as proposed by Gázquez et al. (2007), allows the verification of the possible correlation between these reactivity indices and the RAF antioxidant capability of the investigated molecule. Results are shown in Table 3. Since low values of ω^−^ indicate greater antioxidant activity, the analysis of Table 3 shows how *iso*-moracin C seems to have greater scavenging power in the aqueous environment. On the contrary, in the PE solvent, the antioxidant action of moracin C is greater. Considering the average of the values obtained in the two solvents as previously suggested by some authors (Ceron-Carrasco et al., 2012), values of ω^−^ being very close to each other are obtained (4.36 and 4.38 eV for moracin C and *iso*-moracin C, respectively), making it difficult to reliably predict their correlation with the antioxidant activity of the two molecules. The calculation of the kinetic constants can shed further light on the antioxidant activity of the two systems.

**Table 3:** Ionization potential (IP), electron affinity (AE), electrodonating (ω-), and electroaccepting (ω+) indices of moracin C and *iso*-moracin C in water and PE (in parentheses) environments.

**Molecule**	**IP**	**AE**	**ω^−^**	**ω^+^**
Moracin C (H_3_A)	5.04 (4.98)	1.32 (1.12)	4.54 (4.18)	1.36 (1.13)
Iso-moracin C (H_3_B)	5.14 (4.79)	1.24 (1.25)	4.45 (4.31)	1.26 (1.29)

*All values are in eV*.

Using the data from Table 2 and following the QM-ORSA computational protocol (Galano and Alvarez-Idaboy, 2013), we computed the individual, as well as the total kinetic, constants that are reported in Table 4.

**Table 4:** Rate constants (M^−1^s^−1^) and branching ratios (Γ) computed at the M05-2x level of theory at 298.15 K, **(A)** in aqueous and **(B)** in pentyl ethanoate solvent.

	**H_**3**_A**		**H_**2**_A^**−**^**		**H_**3**_B**		**H_**2**_B^**−**^**	
**Mechanism**	**k (M^**−1**^s^**−1**^)**	**Γ (%)**	**k (M^**−1**^s^**−1**^)**	**Γ (%)**	**k (M^**−1**^s^**−1**^)**	**Γ (%)**	**k (M^**−1**^s^**−1**^)**	**Γ (%)**
**(A)**								
SET	1.03 × 10^−8^	~0.00	1.83 × 10^9^	100.0	1.08 × 10^−9^	~0.00	8.23 × 10^8^	100.0
HAT-O6	4.57 × 10^2^	~0.00	4.45 × 10^2^	~0.0	2.45 × 10^2^	~0.00	9.74 × 10^2^	~0.0
HAT-O3'			7.49 × 10^7^	~0.0	2.79 × 10^9^	~0.00		
HAT-O5'	7.49 × 10^1^	~0.00			4.39 × 10^−2^	~0.00	3.97 × 10^1^	~0.00
HAT-C1″	2.99 × 10^2^	~0.00						
HAT-C3″					1.09 × 10^3^	~0.00		
RAF-C2″	2.15 × 10^9^	100.00			2.15 × 10^9^	100.00		
Total	2.15 × 10^9^		1.83 × 10^9^		2.15 × 10^9^		8.23 × 10^8^	
Overall	2.11 × 10^9^		2.93 × 10^7^		2.13 × 10^9^		9.88 × 10^6^	
	**H**_**3**_**A**^**PE**^		**H**_**3**_**B**^**PE**^					
**Mechanism**	**k (M**^**−1**^**s**^**−1**^**)**	**Γ** **(%)**	**k (M**^**−1**^**s**^**−1**^**)**	**Γ** **(%)**				
**(B)**								
HAT-O6	8.71 × 10^2^	~0.00	1.94 × 10^4^	~0.00				
HAT-O5'			3.42 × 10^1^	~0.00				
HAT-C1″	2.88 × 10^9^	56.68						
HAT-C3″			1.57 × 10^6^	0.07				
RAF-C2″	2.20 × 10^9^	43.32	2.22 × 10^9^	99.93				
Total	5.08 × 10^9^		2.22 × 10^9^					
Overall	4.98 × 10^9^		2.20 × 10^9^					

For neutral moracin C (H_3_A) in the water medium, the faster process is the RAF mechanism in the C2″ site (k = 2.15 × 10^9^ M^−1^s^−1^; branching ratio Γ = 100%), while for the corresponding monoanion (H_2_A^−^), the SET mechanism is the faster process (k = 1.83 × 10^9^ M^−1^s^−1^, branching ratio Γ = 100%). In the *iso*-moracin neutral system (H_3_B), the calculated kinetic constants for RAF and HAT mechanisms are similar (k = 2.79 × 10^9^ M^−1^s^−1^ for HAT on the O3′ site and k = 2.15 × 10^9^ M^−1^s^−1^ for RAF on the C2″ atom), but the branching ratio for the former is 100%. For both the H2X- species, the SET mechanism is preferred (k = 1.83 × 10^9^ M^−1^s^−1^ for moracin and 8.23 × 10^8^ M^−1^s^−1^ for *iso*-moracin).

In the PE environment, for H_3_B, RAF is the preferred mechanism on the C2″ site with a k value of 2.22 × 10^9^ M^−1^s^−1^, while, for H_3_A, a competition between RAF on C2″ (k = 2.20 × 19^9^ M^−1^s^−1^; Γ = 43.32%) and HAT on the C1″ site (k = 2.88 × 19^9^ M^−1^s^−1^; Γ = 56.68%) mechanisms was found.

From the obtained individual and total kinetic constants, it is clear that the experimental mass spectrometric suggestion, according to which the RAF mechanism is best possible solution (Li et al., 2018), is theoretically confirmed. The data indicate that the scavenging activity in the water solution of both moracin C and *iso*-moracin neutral forms is carried out through the RAF mechanism. In the lipid-like environment, the situation appears to be different since mainly the non-redox RAF reaction on C3″ site can occur through the OOH attacking *iso*-moracin C, while moracin C can undergo the attack through both redox (SET)- and non-redox- (RAF) like reactions.

## Conclusion

From the density functional computations on the antioxidant potential of moracin C and its isomer *iso*-moracin C, the following conclusions can be outlined:

-pKa calculations in water environment evidence that, for both systems, the neutral form is dominant with the monoanionic species being present in lower percentage but not negligible;
-the preferred atomic sites for the different reaction mechanisms were established;
-the attack of the OOH free radical, for both the isomers, on the most abundant neutral species in a water solvent mainly occurs through a radical addition mechanism;
-for *iso*-moracin C, the radical addition process is favored in the lipid-like environment, while, for moracin C, both redox- and non-redox-type reactions can occur equally.

## Data Availability Statement

The original contributions presented in the study are included in the article/Supplementary Materials, further inquiries can be directed to the corresponding author.

## Author Contributions

AP, BD, TM, MT, and NR made equal contributions to the study and the publication of this work. All authors contributed to the article and approved the submitted version.

## Conflict of Interest

The authors declare that the research was conducted in the absence of any commercial or financial relationships that could be construed as a potential conflict of interest.
