# Supplementary material for: Quantum Mechanical Predictions of the Antioxidant Capability of Moracin C Isomers
Source: Front Chem. 2021 Apr 21;9:666647. doi: 10.3389/fchem.2021.666647 (PMC8097241; doi:10.3389/fchem.2021.666647)
Supplement: Supplementary file 1 [file Data_Sheet_1.docx]

Supplementary Material

- **Quantum mechanical predictions of the antioxidant capability of moracin C isomers**

**Angela Parise^1,2^, Bruna Clara De Simone^1^, Tiziana Marino^1^, Marirosa Toscano^1^, Nino Russo^1*^**

1 Dipartimento di Chimica e Tecnologie Chimiche, Università della Calabria, 87036 Rende, Italy

2 Université Paris-Saclay, CNRS, Institut de Chimie Physique UMR8000, Orsay, France

*** Correspondence:**Nino Russo

nrusso@unical.it

Supplementary Data


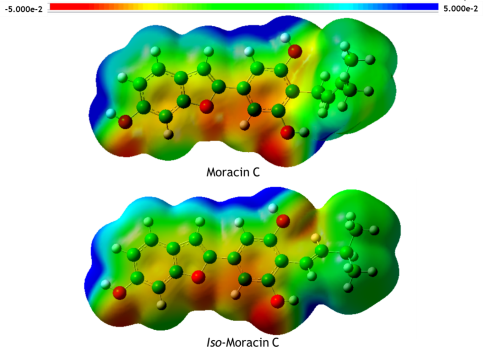


Figure S1. Molecular electrostatic potential (MEP) for moracin C and i*so*-moracin C structures optimized in water at M05-2X/6-311++G(d,p) level of theory.


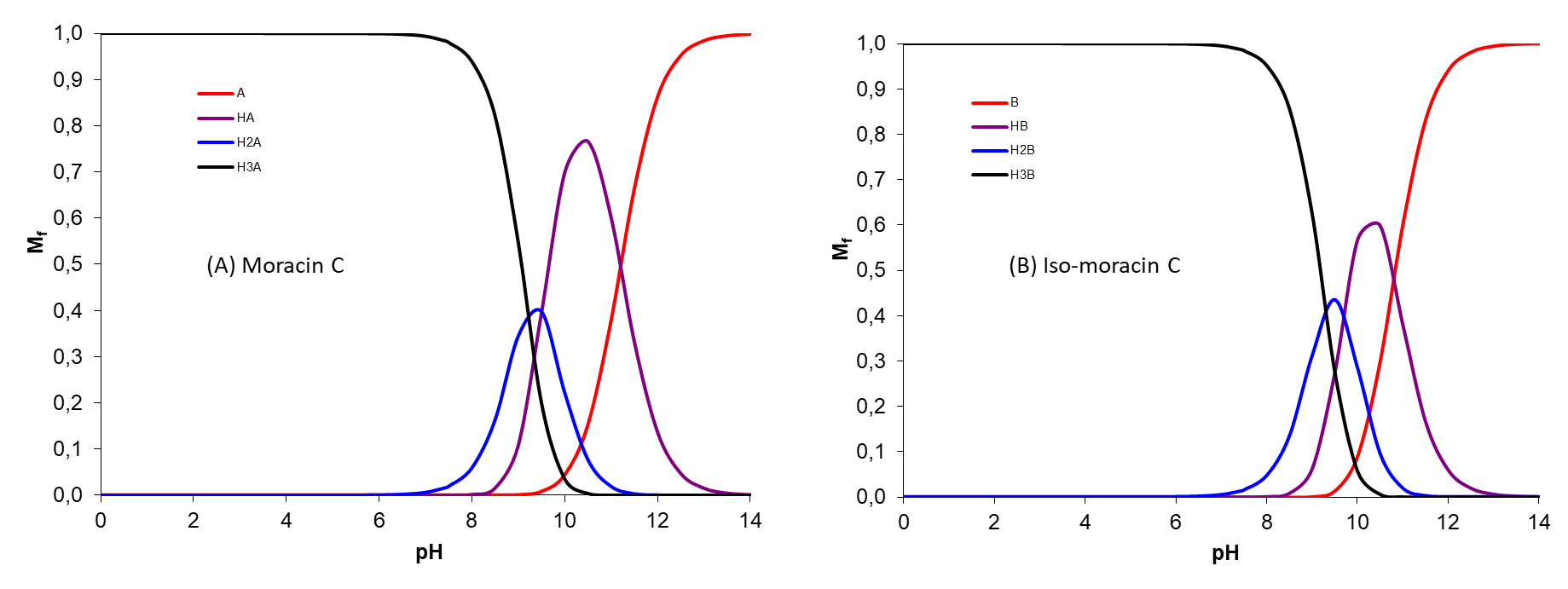


Figure S2. Molar fractions (M f) of the different acid–base species of moracin C and *iso*-moracin C at physiological pH.


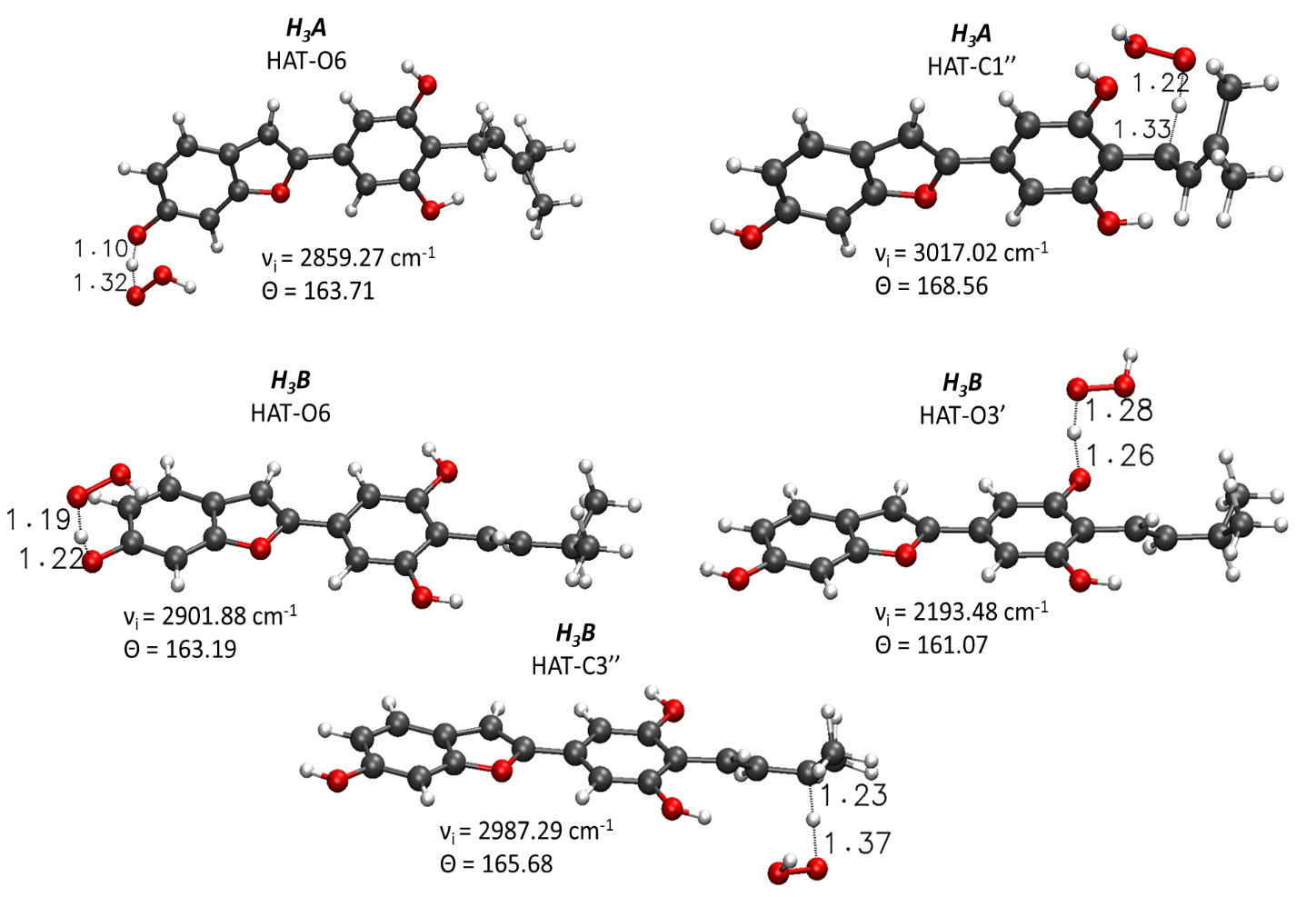


Figure S3. Main geometrical parameters for the optimized TSs structures in lipidic environment for the HAT mechanism of neutral moracin C and *iso*-moracin C. Bond lengths are in Å, angles in degrees and imaginary frequencies in cm-1.


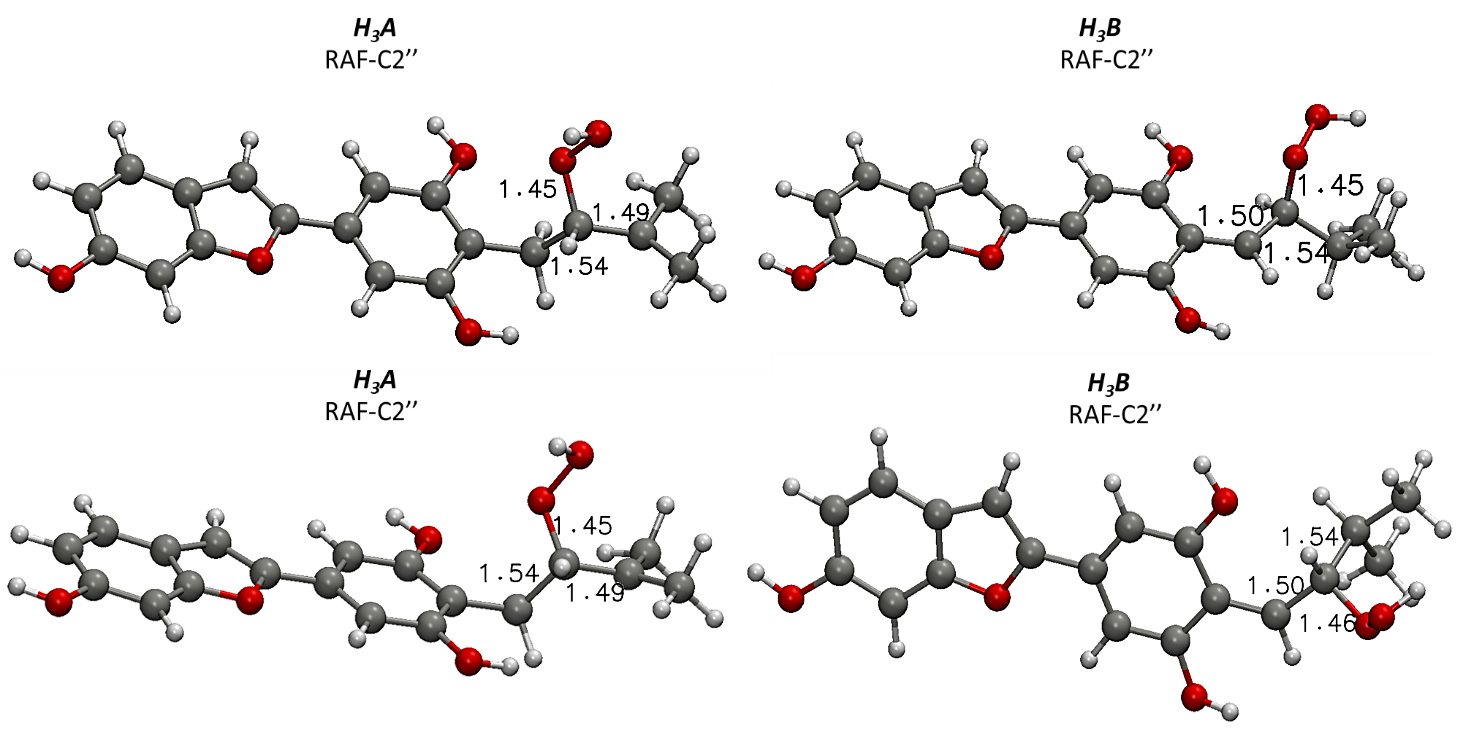


**Figure S4**.. Main geometrical parameters for the optimized structures in water (on top) and lipidic (down) solvent, concerning the RAF mechanism, for the OOH-moracin C and OOH-iso-moracin C. Bond lengths are in Å.

**Table S1.** NBO charge values in |e| for the optimized geometries of moracin C and *iso*-moracin C.

| **NBO** | Moracin C | Iso-moracin C |
| --- | --- | --- |
| O (6) | -0.761 | -0.761 |
| H (6) | 0.552 | 0.552 |
| O (3’) | -0.758 | -0.754 |
| H (3’) | 0.556 | 0.554 |
| O (5’) | -0.756 | -0.756 |
| H (5’) | 0.556 | 0.552 |
| C (4’) | -0.152 | -0.193 |
| C (1’’) | -0.533 | -0.301 |
| C (2’’) | -0.266 | -0.170 |
| C (3’’) | -0.029 | -0.339 |
| C (4’’) | -0.743 | -0.704 |
| H1 (C4’’) | 0.261 | 0.244 |
| H2 (C4’’) | 0.255 | 0.251 |
| H3 (C4’’) | 0.260 | 0.252 |
| C (5’’) | -0.733 | -0.719 |
| H1 (C5’’) | 0.257 | 0.246 |
| H2 (C5’’) | 0.257 | 0.251 |
| H3 (C5’’) | 0.255 | 0.256 |

**Table S2.** CC bond lengths and atomic spin density values for the OOH addition to the C2” atom of Moracin C and Iso-Moracin C in water (A) and PE (B) environments

|  | **Water** | | | | **PE** | | | |
| --- | --- | --- | --- | --- | --- | --- | --- | --- |
|  | **H_3_A RAF-C2''** | | **H_3_B RAF-C2''** | | **H_3_A RAF-C2''** | | **H_3_B RAF-C2''** | |
| **Atom** | **d (Å)** | **Spin density** | **d (Å)** | **Spin density** | **d (Å)** | **Spin density** | **d (Å)** | **Spin density** |
| 1 | - | 3.60 10^-05^ | - | 1.81 10 ^-03^ | - | 3.60 10 ^-05^ | - | 4.31 10 ^-03^ |
| 8 | 1.361 | 2.34 10^-04^ | 1.369 | 1.24 10 ^-02^ | 1.371 | 2.34 10 ^-04^ | 1.369 | 2.83 10 ^-02^ |
| 7 | 1.395 | -1.64 10 ^-04^ | 1.384 | -8.52 10 ^-03^ | 1.394 | -1.64 10 ^-04^ | 1.381 | -2.00 10 ^-02^ |
| 6 | 1.394 | 1.99 10 ^-04^ | 1.392 | 1.40 10 ^-02^ | 1.394 | 1.99 10 ^-04^ | 1.390 | 3.30 10 ^-02^ |
| 5 | 1.410 | -6.50 10 ^-05^ | 1.411 | -5.02 10 ^-03^ | 1.409 | -6.50 10 ^-05^ | 1.411 | -1.22 10 ^-02^ |
| 4 | 1.391 | 2.95 10 ^-04^ | 1.392 | 1.49 10 ^-02^ | 1.382 | 2.95 10 ^-04^ | 1.393 | 3.64 10 ^-02^ |
| 9 | 1.460 | -2.01 10 ^-04^ | 1.460 | -1.63 10 ^-02^ | 1.403 | -2.01 10 ^-04^ | 1.403 | -3.70 10 ^-02^ |
| 3 | 1.441 | 1.22 10 ^-03^ | 1.443 | 5.25 10 ^-02^ | 1.441 | 1.22 10 ^-03^ | 1.440 | 1.25 10 ^-01^ |
| 2 | 1.351 | -5.32 10 ^-04^ | 1.357 | -4.26 10 ^-02^ | 1.357 | -5.32 10 ^-04^ | 1.361 | -9.33 10 ^-02^ |
| 1' | 1.459 | 3.23 10 ^-03^ | 1.453 | 1.17 10 ^-01^ | 1.462 | 3.23 10 ^-03^ | 1.451 | 2.46 10 ^-01^ |
| 2' | 1.403 | 2.45 10 ^-03^ | 1.401 | -7.92 10 ^-02^ | 1.403 | 2.45 10 ^-03^ | 1.411 | -1.45 10 ^-01^ |
| 3' | 1.392 | -1.35 10 ^-03^ | 1.382 | 1.26 10 ^-01^ | 1.381 | -1.35 10 ^-03^ | 1.384 | 2.60 10 ^-01^ |
| 4' | 1.404 | -2.13 10 ^-02^ | 1.441 | -1.03 10 ^-01^ | 1.402 | -2.13 10 ^-02^ | 1.439 | -2.75 10 ^-01^ |
| 1'' | 1.508 | 1.33 10 ^-02^ | 1.400 | 8.85 10 ^-01^ | 1.503 | 1.33 10 ^-02^ | 1.403 | 7.44 10 ^-01^ |
| 2'' | 1.543 | -1.59 10 ^-02^ | 1.504 | -3.79 10 ^-02^ | 1.544 | -1.59 10 ^-02^ | 1.499 | -8.85 10 ^-02^ |
| 3'' | 1.513 | 9.26 10 ^-01^ | 1.541 | 3.04 10 ^-03^ | 1.491 | 9.26 10 ^-01^ | 1.542 | 2.64 10 ^-02^ |
| 4'' | 1.491 | 3.23 10 ^-02^ | 1.530 | 1.51 10 ^-03^ | 1.490 | 3.23 10 ^-02^ | 1.531 | -2.67 10 ^-03^ |
